# Shared decision-making practices and patient values in pharmacist outpatient care for rheumatic disease: A multiple correspondence analysis

**DOI:** 10.3389/jpps.2023.11135

**Published:** 2023-01-20

**Authors:** Ikkou Hirata, Shunsuke Hanaoka, Ryo Rokutanda, Ryohkan Funakoshi, Hiroyuki Hayashi

**Affiliations:** ^1^ Department of Pharmacy, Kameda General Hospital, Chiba, Japan; ^2^ Department of Clinical Pharmacotherapy, School of Pharmacy, Nihon University, Chiba, Japan; ^3^ Department of Rheumatology, Kameda General Hospital, Chiba, Japan

**Keywords:** rheumatic disease, shared decision-making, patient values, pharmacist outpatient care, multiple correspondence analysis

## Abstract

**Purpose:** To investigate the value-to-value relationships, relationship between values and patient background, continuation rate of treatment after shared decision-making (SDM), and disease status in order to clarify the values involved in drug therapy decisions for patients with rheumatic disease.

**Methods:** We investigated patient values (efficacy of drug therapy [effectiveness], safety, economics, daily life, and other) and the continuance rate and disease status of treatment after 6 months in 94 patients with rheumatic disease aged ≥18 years who made decisions with pharmacists and physicians in the pharmacy outpatient clinic between September 2019 and April 2021. Multiple correspondence and K-means cluster analyses were performed to show the relationship between values and basic patient information.

**Results:** Among the selected patients, 87% and 47% selected effectiveness for multiple selections and single selection, respectively. Effectiveness was at the center of the graph; three clusters containing other values were placed around it. History of allergy or side effects caused by biologics or Janus kinase inhibitors were in the safety cluster. The non-usage history of biologics or Janus kinase inhibitors was in the economic cluster.

**Conclusion:** Effectiveness was the most important factor for patients with rheumatic disease; the values that patients consider important may shift from effectiveness to other values based on each patient’s subjective experience with the treatment and/or the stage of life in which they were treated. It is important to positively link patient values and information about the treatment plan in shared decision-making while establishing rapport with the patient.

## Introduction

In recent years, shared decision-making (SDM) practices are regarded as important when initiating or changing treatment (1, 2). SDM is a communication process between a patient and a healthcare provider that integrates evidence-based medicine with patient values and emotions. Patients and physicians are required to implement SDM as the minimum combination (1–5). However, there are reports of pharmacists participating in SDM for treatment of psychiatric, diabetic, and cardiovascular diseases (6–9).

Rheumatic diseases, such as rheumatoid arthritis (RA) and other connective tissue diseases, are mostly treated by drug therapy. Therefore, adherence is important, and patient participation in drug therapy is essential (2, 10). Physicians’ drug decisions are based on their expectations of therapeutic efficacy. Pharmacists, in contrast, determine the suitability of drugs for patients by considering various factors, such as adverse events and side effects. Implementing SDM with patients involving collaboration between physicians and pharmacists with different perspectives on drugs leads to concordance. The patient participates as a member of the medical care team, which may improve patient adherence and solve the effectiveness, safety, economic, and daily life issues in drug therapy.

In clinical practice, favorable decision-making for patients and high-quality drug therapies are achieved when pharmacists are involved in SDM. However, there is no report on the analysis of the values that patients use as criteria for the selection of drug therapies when SDM involves pharmacists. Therefore, we investigated the value-to-value relationships, relationship between values and patient background, continuation rate of treatment after SDM, and disease status in order to clarify the values involved in drug therapy decisions for patients.

## Materials and methods

### Study design

This was a single-center, cross-sectional, retrospective observational study. This study complied with the standards of the Declaration of Helsinki and the current ethical guidelines. The design and methodology, including the opt-out method of consent available to all patients, were approved by the Kameda General Hospital Clinical Research Review Committee (approval number: 21-010).

### SDM process

**FIGURE 1:**
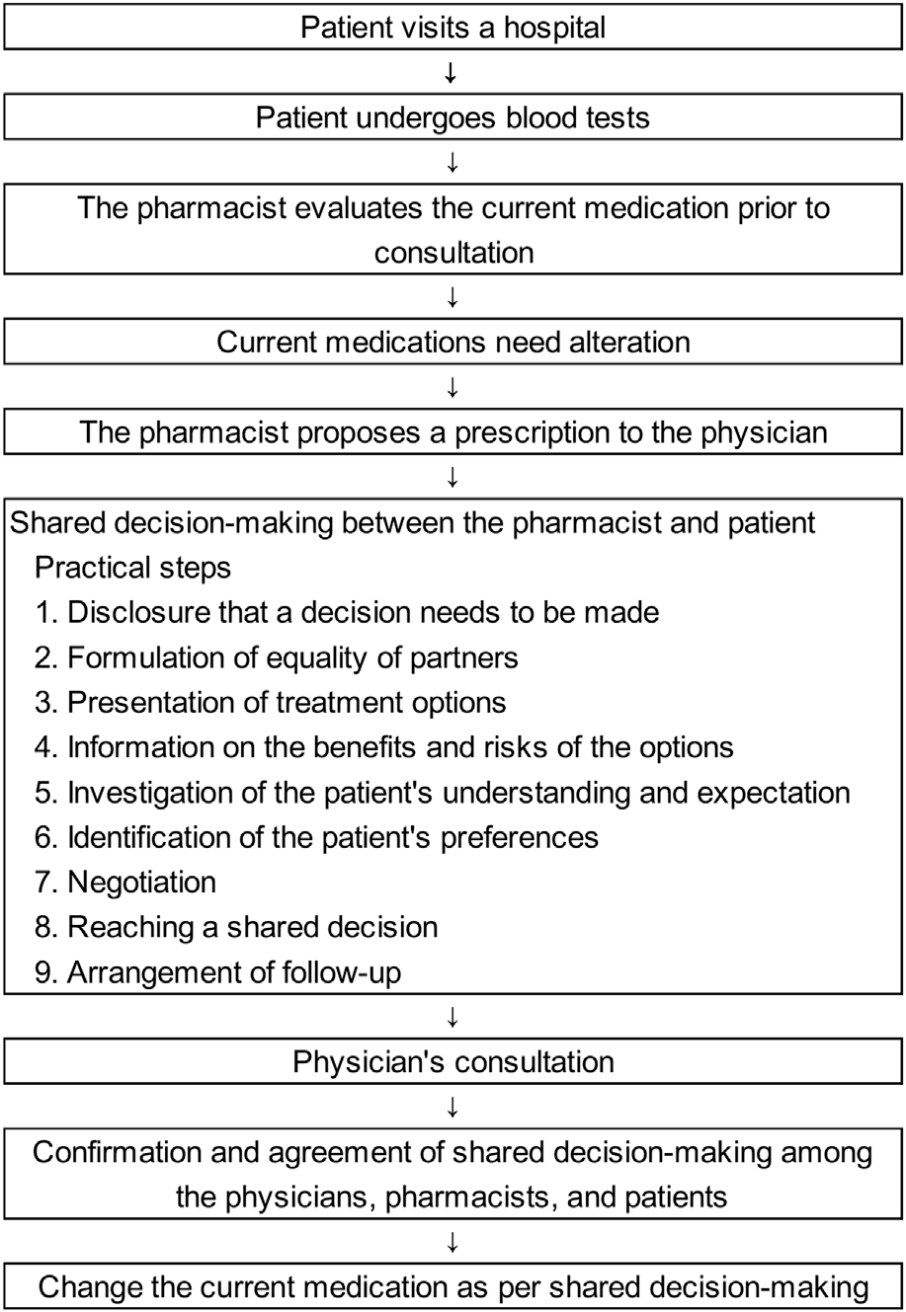
Shared decision-making process and flow.

The process and flow for SDM are shown in Figure 1 (3, 4). In 2015, Kameda General Hospital started a pharmacist outpatient clinic in the inflammatory bowel disease specialty outpatient clinic and now also offers this service in the outpatient clinic of the rheumatism, collagen disease, allergy, internal medicine department. In this pharmacist outpatient clinic, pharmacists participate in SDM and provide support in situations in which drug therapy needs to be intensified or changed. All patients with rheumatic disease received medical consultations and were treated according to the guidelines from Phase 1 onwards. The pharmacist evaluated the current medications of all patients prior to the physician’s consultation during their regular clinical practice regardless of the duration of the disease. Patients whose condition was stable proceeded directly to the physician’s consultation. For patients considered by the pharmacist to be likely to have difficulty continuing drug treatment, or patients who requested a change in drug therapy, an SDM was made between the pharmacist and patient according to SDM-Q-9 (11).

To present patients with new treatment options that are consistent with their values in SDM, pharmacists confirmed the values that the patient considered important in selecting drug treatment with the patients. The pharmacist presented five values to the patient: the efficacy of drug therapy (effectiveness), the safety of drug therapy (safety), the economic burden (economical), the impact of drug treatment on the quality of life (daily life), and other physical and mental burdens caused by drug treatment (other). The “economical” value was defined as a patient’s comprehensive view of the cost of drugs relative to their income at a specific stage of their life. “Daily life” refers to the impact of treatment on daily life (routine living and habits), such as the frequency of hospital visits. “Other” factors included the physical and mental burden of drug treatment on the patient, such as the type of device used, route of administration, and duration of intravenous infusion. Patients initially made several selections from the five values influencing their treatment choice (multiple selections) and subsequently chose one value as the most important to prioritize during treatment selection (single selection). The pharmacist suggested that the value selected by the patient as most important be resolved first, and also provided information to help resolve the other multiple-selected values. After the pharmacist and patient shared a new treatment decision, the pharmacist proposed a prescription based on the results of the SDM to the physician. The physician examined the patient, and they were given the opportunity to reject the SDM results, which was agreed upon in advance by the pharmacist and patient. The physician, pharmacist, and patient consulted again to confirm no difficulties with the previously determined SDM, and the current medications were changed.

### Study population

We retrospectively enrolled all 94 patients with rheumatic disease aged ≥18 years who were involved in decision-making with a pharmacist and physician in the pharmacist outpatient clinic between September 2019 and April 2021. If one patient attends more than one SDM session, each SDM session was enrolled as one patient because the values that patients select for each SDM session can change with disease status, life stage changes, and other factors. Rheumatic diseases included RA, adult-onset Still’s disease, ankylosing spondylitis, Behcet’s disease, systemic lupus erythematosus, spondylarthritis, psoriasis, psoriatic arthritis, Sjogren’s syndrome, and palmoplantar pustulosis.

### Survey items

Patient information at the time of SDM implementation was retrospectively obtained from the medical records. The surveyed items were sex, age (years), age (<65 years/≥65 years), RA, multiple rheumatic diseases, disease duration (years), disease duration (<10/≥10 years), disease activity (active/inactive), number of drugs used (number), number of drugs used (<5/≥5), history of allergy or side effects, history of allergy or side effects due to biologics or Janus kinase inhibitor, biologics, or Janus kinase inhibitor usage history before SDM. Age was classified into two categories according to the World Health Organization (WHO). Disease duration was classified into two categories based on a previous report on RA (12). Disease activity was classified into two categories; patients with RA were categorized as inactive if they exhibited low disease activity or remission based on their DAS28-CRP value and as active if they exhibited moderate or high disease activity. Those with other rheumatic diseases were classified as inactive if clinically judged by a physician to be in remission or have low disease activity and as active if they were otherwise judged to have moderate or higher-intensity symptoms. The number of drugs used was classified into two categories based on a previous report on polypharmacy (13).

The influential values of patients regarding drug treatment were compiled from subjective data describing values chosen by patients during conversations between pharmacists and patients during SDM implementation. The values compiled were multiple selected and single selected from the five values: effectiveness, safety, economic, daily life (drug treatment burden on life), and others (such as route of administration, type of device).

The continuance rate of treatment 6 months after SDM and disease status (improvement, aggravation, and no change) were evaluated, with reasons including inadequate effectiveness, side effects, economic issues, daily life issues, and other issues among patients.

The details of the drug treatment changes selected after the implementation of SDM were tabulated. The results included changing the drugs, tight control, no change, drug use cessation, increased dosage, change in the route of administration, addition of an oral drug, oral drug cessation, reduced dosage, changes in oral drug, and shortening of the interval between doses.

Because SDM was performed in the usual clinical setting, the pharmacists were not blinded to the outcomes, such as the continuance rate of treatment.

### Statistical analysis

Regarding basic information on patient characteristics, patient selection of SDM values, treatment continuity, and disease activity after SDM, continuous variables were expressed as the median (interquartile range), and categorical variables were expressed as numbers (%). Fisher’s exact tests were performed to compare categorical variables, and Mann–Whitney U tests were performed to compare continuous variables between the two groups. *p* < 0.05 was considered statistically significant.

Multivariate analysis was performed using multiple correspondence analysis (MCA) to show the relationship between values and basic patient characteristics. MCA is an extension of correspondence analysis when multiple variables are considered, and a method of analyzing categorical/categorized data and presenting the results in a graph (map). The categorical variables that were fed into MCA and transformed into a cross table are listed below (14).

#### Patient values involved in SDM

Influential multiple and most important single selections values with yes/no response consisted of effectiveness, safety economy, daily life and others.

#### Basic information on patient characteristics

Sex (female or male), age_group (<65/≥65 years), disease duration_group (<10/≥10 years), disease activity (active/inactive), number of drugs used_group (<5/≥5), history of allergy or side effects caused by drugs in general (yes/no), history of allergy or side effects caused by biologics or Janus kinase inhibitor (yes/no), biologics or Janus kinase inhibitor usage history_before SDM (yes/no).

The information described in each dimension was evaluated using the Greenacre inertia adjustment, and the categorical variables were plotted in two dimensions with the highest inertia (15). A K-means cluster analysis, a non-hierarchical cluster analysis identifying mutually exclusive clusters by calculating the quadratic Euclidean distance (coefficient of similarity) of the point categories (16, 17), was necessary to objectively ascertain which values each of the patient background items related to or belonged to. The coordinates (object scores) of each of the dimensions 1 and 2 of each variable calculated by MCA were input to K-means cluster analysis and the categorical variables, including the values and the basic information of patient characteristics, were grouped (18). The cubic clustering criterion (CCC) was calculated with statistical software and used to determine the optimal number of clusters (19, 20). After clustering each variable, density ellipses (*α* = 95%) for each cluster were shown to indicate the overlap between clusters and were overlaid with the MCA plot. Statistical analyses were performed using JMP Pro 16 software (SAS Institute Inc., Cary, NC, United States).

## Results

### Patient characteristics

The patient characteristics are shown in Table 1. Ninety-four patients underwent SDM with a pharmacist during the study period. The median patient age was 66 [52–71] years, and 70% were women. Among these patients, 89% had RA and 11% had other rheumatic diseases as primary rheumatic disease. Nine percent of all patients were affected by multiple rheumatic diseases. Patient acceptance of the results of SDM in collaboration with physicians and pharmacists was 98%, with only two refusals (one patient and one physician).

**TABLE 1:** Patient characteristics.

	n or median	(%) or [range]
Overall	94	(100)
Age (years)	66	[52–71]
Age (≥65 years)	48	(51)
Sex (female)	66	(70)
Primary rheumatic disease		
RA	84	(89)
Other rheumatic diseases	10	(11)
Patients affected by multiple rheumatic diseases	8	(9)
Disease duration (years)	8	[3–6]
Disease duration (≥10 years)	40	(43)
Disease activity (active)	66	(70)
Number of drugs used	6	[4–8]
Number of drugs used (≥5)	64	(68)
Number of BIO or JAK usage history before SDM	1	[1–2]
BIO or JAK usage history before SDM	72	(77)
History of allergy or side effects	45	(48)
History of allergy or side effects (BIO or JAK)	24	(26)
Patient rejection	1	(1)
Physician rejection	1	(1)

Other rheumatic diseases included adult-onset Still’s disease, ankylosing spondylitis, Behcet’s disease, systemic lupus erythematosus, spondylarthritis, psoriasis, psoriatic arthritis, Sjogren’s syndrome, and palmoplantar pustulosis. Categorical variables were expressed as numbers (%), and continuous variables were expressed as the median [interquartile range]. RA, rheumatoid arthritis; BIO, biologics; JAK, Janus kinase inhibitor; SDM, shared decision-making.

### Influential values involved in SDM

The values that the patients selected as important in their decision-making regarding drug therapy are shown in Table 2. Among eligible patients, 87% and 47% selected effectiveness for multiple selections and single selections, respectively: most patients selected effectiveness. Therefore, effectiveness was the most important influential factor. Out of 25 patients who selected “other” for multiple selections, 20 marked the route of administration as a factor (data not shown).

**TABLE 2:** Values involved in shared decision-making.

Values	n	(%)
Overall	94	(100)
Influential values (multiple selections)		
Effectiveness_multiple_yes	82	(87)
Safety_multiple_yes	54	(57)
Economical_multiple_yes	30	(32)
Daily life_multiple_yes	23	(24)
Other_multiple_yes	25	(27)
Most important influential values (single selection)		
Effectiveness_most	44	(47)
Safety_most	24	(26)
Economical_most	14	(15)
Daily life_most	3	(3)
Other_most	9	(10)

Patients initially made several selections from the five values influencing their treatment choice (multiple selections, “multiple_yes”) and subsequently chose one value as the most important to be prioritized during treatment selection (single selection, “most”). Categorical variables are expressed as numbers (%).

### Analysis of influential values (multiple selections) compared with the most important values (single selection)

The patient characteristics and values selected in multiple selection were compared for each value selected in a single selection (Table 3). Age (years) (*p* = 0.001), age (group) (*p* = 0.008), and disease activity (active) (*p* = 0.001) showed significant differences. In the multiple selections of values, significant differences were observed. Moreover, a comparison of patient characteristics between the two disease activity groups revealed that the Active group had a significantly had a lower history of biologics or Janus kinase inhibitor use prior to SMD than did the Inactive group (68% vs. 96%, *p* = 0.003), although there were no significant between-group differences in the remaining items (data not shown).

**TABLE 3:** Results of a single regression analysis of patient characteristics and influential values (multiple selections) compared with the most important influential values (single selection).

Patient characteristics and influential values (multiple selections)	Overall		Most important influential values (single selection)										*p*
			Effectiveness_most		Safety_most		Economical_most		Daily life_most		Other_most		
	n or median	(%) or [range]	n or median	(%) or [range]	n or median	(%) or [range]	n or median	(%) or [range]	n or median	(%) or [range]	n or median	(%) or [range]	
Overall	94	(100)	44	(100)	24	(100)	14	(100)	3	(100)	9	(100)	–
Patient characteristics													
Age (years)	66	[52–71]	66	[53–71]	66	[53–71]	52	[42–57]	78	[78–78]	73	[63–78]	0.001
Age (≥65 years)	48	(51)	23	(52)	13	(54)	2	(14)	3	(100)	7	(78)	0.008
Sex (female)	66	(70)	32	(73)	18	(75)	9	(64)	3	(100)	4	(44)	0.360
Disease duration (years)	8	[3–16]	7	[2–14]	8	[3–16]	7	[4–12]	55	[9–55]	14	[6–37]	0.094
Disease duration (≥10 years)	40	(43)	19	(43)	11	(46)	3	(21)	2	(67)	5	(56)	0.399
Disease activity (active)	66	(70)	38	(86)	14	(58)	9	(64)	3	(100)	2	(22)	0.001
Number of drugs used	6	[4–8]	7	[4–9]	5	[4–8]	5	[4–7]	5	[5–13]	7	[6–9]	0.239
Number of drugs used (≥5)	64	(68)	30	(68)	16	(67)	7	(50)	3	(100)	8	(89)	0.307
BIO or JAK usage history before SDM	72	(77)	34	(77)	19	(79)	8	(57)	3	(100)	8	(89)	0.429
History of allergy or side effects (drugs in general)	45	(48)	19	(43)	15	(63)	5	(36)	2	(67)	4	(44)	0.463
History of allergy or side effects (BIO or JAK)	24	(26)	9	(20)	11	(46)	2	(14)	0	(0)	2	(22)	0.133
Influential values (multiple selections)													
Effectiveness_multiple_yes	82	(87)	44	(100)	14	(58)	14	(100)	3	(100)	7	(78)	<0.0001
Safety_multiple_yes	54	(57)	22	(50)	24	(100)	4	(29)	0	(0)	4	(44)	<0.0001
Economical_multiple_yes	30	(32)	11	(25)	3	(13)	14	(100)	0	(0)	2	(22)	<0.0001
Daily life_multiple_yes	23	(24)	11	(25)	0	(0)	4	(29)	3	(100)	5	(56)	<0.0001
Other_multiple_yes	25	(27)	11	(25)	5	(21)	0	(0)	0	(0)	9	(100)	<0.0001

Effectiveness, Safety, Economical, Daily life, and Other are the values that the patients considered important when selecting drug treatment. Effectiveness indicated the efficacy of drug therapy, Safety indicated the safety of drug therapy, Economical indicated the economic burden, Daily life indicated the impact of drug treatment on the quality of life, and Other indicated other physical and mental burdens caused by treatment. Patients initially made several selections from the five values influencing their treatment choice (multiple selections, “multiple_yes”) and subsequently chose one value as the most important to prioritize during treatment selection (single selection, “most”). Categorical variables are expressed as numbers (%), and continuous variables were expressed as the median [interquartile range]. Fisher’s exact tests were performed to compare categorical variables, and Mann–Whitney U tests were performed to compare continuous variables between the two groups. *p* < 0.05 was considered statistically significant. BIO, biologics; JAK, Janus kinase inhibitor; SDM, shared decision-making.

### Analysis of patient characteristics compared with each influential value (multiple selections)

For each of the five values marked on multiple selections, the patient characteristics were compared between the selected (yes) and non-selected (no) groups (Table 4). In terms of effectiveness, the “yes” group had significantly fewer patients aged ≥65 years than the “no” group (*p* = 0.028). With respect to safety, female sex (*p* = 0.024), inactive disease (*p* = 0.039), biologics or Janus kinase inhibitor usage history before SDM (*p* = 0.028), history of allergy or side effects caused by drugs in general (*p* = 0.004), and history of allergy or side effects caused by biologics or Janus kinase inhibitor (*p* < 0.0001) were significantly more frequent in the “yes” group than in the “no” group. Regarding the economics, the “yes” group was significantly younger (*p* = 0.007) and had significantly fewer patients aged ≥65 years (*p* = 0.002). History of biologics or Janus kinase inhibitor use before SDM was significantly lower in the “yes” group (*p* = 0.017). No significant differences were found in any of the items in daily life. The “yes” group was significantly older (*p* = 0.003) and had more patients aged ≥65 years (*p* = 0.001). The disease duration was significantly longer in the “yes” group (*p* = 0.046). Disease activity was significantly higher in the “no” group than in the “yes” group (*p* = 0.039).

**TABLE 4:** Results of single regression analysis of patient characteristics compared with each influential value (multiple selections).

Patient characteristics	Influential values (multiple selections)		*p*
	n (%) or median [range]	n (%) or median [range]	
	**Effectiveness_multiple**		
	**Yes**	**No**	
Overall	82 (100)	12 (100)	—
Age (years)	63 [52–71]	68 [66–75]	0.085
Age (≥65 years)	38 (46)	10 (83)	0.028
Sex (female)	56 (68)	10 (83)	0.500
Disease duration (years)	8 [3–17]	6 [3–15]	0.790
Disease duration (≥10 years)	36 (44)	4 (33)	0.548
Disease activity (active)	60 (73)	6 (50)	0.173
Number of drugs used	6 [4–8]	5 [4–7]	0.346
Number of drugs used (≥5)	56 (68)	8 (67)	1.000
BIO or JAK usage history before SDM	19 (23)	3 (25)	1.000
History of allergy or side effects	44 (54)	5 (42)	0.542
History of allergy or side effects of BIO or JAK	64 (78)	6 (50)	0.070
	**Safety_multiple**		
	**Yes**	**No**	
Overall	54 (100)	40 (100)	—
Age (years)	66 [54–70]	64 [48–72]	0.731
Age (≥65 years)	29 (54)	19 (48)	0.677
Sex (female)	43 (80)	23 (58)	0.024
Disease duration (years)	10 [3–16]	5.5 [1–19]	0.257
Disease duration (≥10 years)	27 (50.0)	13 (33)	0.098
Disease activity (active)	33 (61)	33 (83)	0.039
Number of drugs used	7 [4–8]	5 [4–9]	0.535
Number of drugs used (≥5)	39 (72)	25 (63)	0.374
BIO or JAK usage history before SDM	46 (85)	26 (65)	0.028
History of allergy or side effects	33 (61)	12 (30)	0.004
History of allergy or side effects of BIO or JAK	22 (41)	2 (5)	<0.0001
	**Economical_multiple**		
	**Yes**	**No**	
Overall	30 (100)	64 (100)	—
Age (years)	57 [48–67]	67 [53–73]	0.007
Age (≥65 years)	8 (27)	40 (63)	0.002
Sex (female)	21 (70)	45 (70)	1.000
Disease duration (years)	6 [2–12]	9 [3–18]	0.129
Disease duration (≥10 years)	9 (30)	31 (48)	0.119
Disease activity (active)	19 (63)	47 (73)	0.341
Number of drugs used	6 [4–8]	7 [4–8]	0.467
Number of drugs used (≥5)	17 (57)	47 (73)	0.154
BIO or JAK usage history before SDM	18 (60)	54 (84)	0.017
History of allergy or side effects	11 (37)	34 (53)	0.184
History of allergy or side effects of BIO or JAK	5 (17)	19 (29)	0.212
	**Daily life_multiple**		
	**Yes**	**No**	
Overall	23 (100)	71 (100)	—
Age (years)	67 [56–76]	64 [52–71]	0.191
Age (≥65 years)	13 (57)	35 (49)	0.634
Sex (female)	15 (65)	51 (72)	0.604
Disease duration (years)	6 [1–17]	8 [3–16]	0.348
Disease duration (≥10 years)	8 (345)	32 (45)	0.470
Disease activity (active)	17 (74)	49 (69)	0.795
Number of drugs used	8 [5–9]	5 [4–8]	0.070
Number of drugs used (≥5)	18 (78)	46 (65)	0.306
BIO or JAK usage history before SDM	18 (78)	54 (76)	1.000
History of allergy or side effects	12 (52)	33 (47)	0.811
History of allergy or side effects of BIO or JAK	4 (17)	20 (28)	0.413
	**Other_multiple**		
	**Yes**	**No**	
Overall	25 (100)	69 (100)	—
Age (years)	68 [66–75]	60 [49–70]	0.003
Age (≥65 years)	20 (80)	28 (41)	0.001
Sex (female)	16 (64)	50 (73)	0.452
Disease duration (years)	11 [5–18]	7 [3–14]	0.046
Disease duration (≥10 years)	15 (60)	25 (36)	0.058
Disease activity (active)	13 (52)	53 (77)	0.039
Number of drugs used	8 [6–9]	5 [4–8]	0.076
Number of drugs used (≥5)	20 (80)	44 (64)	0.210
BIO or JAK usage history before SDM	23 (92)	49 (71)	0.051
History of allergy or side effects	14 (56)	31 (45)	0.361
History of allergy or side effects of BIO or JAK	5 (20)	19 (28)	0.595

Patients initially made several selections from the five values influencing their treatment choice (multiple selections, “multiple_yes”) and subsequently chose one value as the most important to be prioritized during treatment selection (single selection, “most”). Categorical variables were expressed as numbers (%), and continuous variables were expressed as the median [interquartile range]. Fisher’s exact tests were performed to compare categorical variables, and Mann–Whitney U tests were performed to compare continuous variables between the two groups. *p* < 0.05 was considered statistically significant. BIO, biologics; JAK, Janus kinase inhibitor; SDM, shared decision-making.

### Continuity of treatment and disease status 6 months after SDM

The treatment continuance rate, reasons for treatment cessation, and disease status 6 months after SDM are shown in Table 5. Among the patients, 78% continued with treatment 6 months after SDM, and 90% had either improved or reported no change in disease status. There were no cases of non-adherence or abrupt cessation of treatment. Regardless of the outcome, all patients who had participated in SDM were satisfied with the process.

**TABLE 5:** Continuation of treatment and disease status at 6 months after shared decision-making.

Continuation of treatment and disease status	n	(%)
Overall	94	(100)
Continuity of treatment	73	(78)
Treatment cessation	21	(22)
Inadequate effectiveness	11	(12)
Side effects	10	(11)
Economic issues	2	(2)
Daily life issues	0	(0)
Other issues	0	(0)
Disease status		
Improvement	55	(59)
No change	30	(32)
Aggravation	9	(10)

Categorical variables are expressed as numbers (%).

### Details of change and disease status after SDM

The details of the treatment changes due to SDM are presented in Table 6. Among the patients, 35% changed the route of administration and 52% changed the drugs.

**TABLE 6:** Route of administration before/after shared decision-making and details of change.

Route of administration and details of change	n	(%)
Overall	94	(100)
Route of administration before SDM		
Oral	42	(45)
Subcutaneous self-injection	28	(30)
Intravenous	15	(16)
Subcutaneous injection by a nurse	9	(10)
Route of administration after SDM		
Oral	32	(34)
Subcutaneous self-injection	31	(33)
Subcutaneous injection by a nurse	13	(14)
Intravenous	6	(6)
None	12	(13)
Details of change of administration route		
No change	49	(52)
Change of administration route	33	(35)
Subcutaneous self-injection	14	(15)
Subcutaneous injection by a nurse	9	(10)
Oral	9	(10)
Intravenous	1	(1)
Drug use cessation	12	(13)
Details of treatment change		
Change of drugs^a^	49	(52)
Change of drug	22	(23)
Tight control	22	(23)
Addition of oral drug	2	(2)
Oral drug cessation	2	(2)
Change of oral drug	1	(1)
No change^b^	17	(18)
Drug use cessation	12	(13)
Increased dosage	10	(11)
Change of administration route^c^	3	(3)
Reduced dosage	2	(2)
Shortening of interval between doses	1	(1)

^a^ “Change of drugs” is the sum of the numbers of change of drug, tight control, and addition of oral drug, oral drug cessation, and change of oral drug.

^b^ “No change” signifies that there was no change in treatment. Current treatment was re-selected after SDM was performed.

^c^ “Change of administration route” means that the patients did not change the drug and only changed the administration route. Categorical variables are expressed as numbers (%). SDM, shared decision-making.

### MCA and K-means cluster analysis outcome

**FIGURE 2:**
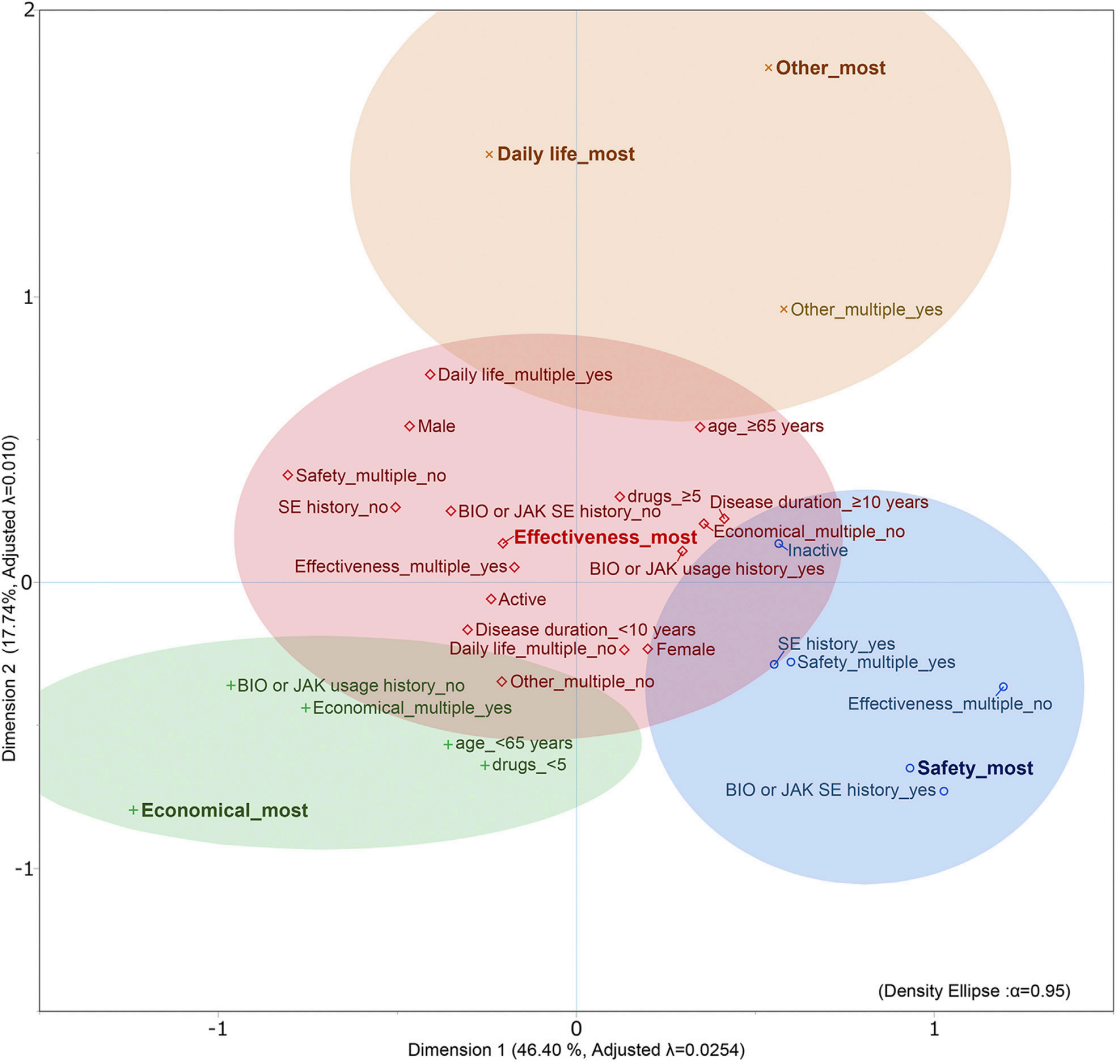
Outcome of MCA and K-means cluster analysis of patients with rheumatic disease. Adjusted λ, Greenacre’s adjusted inertia; BIO, biologics; JAK, Janus kinase inhibitor; most, single selection; multiple, multiple selections; SE, side effects; SE history, history of allergy or side effects.

To conduct MCA, a multidimensional contingency table of all two-way cross-tabulations across all variables, called the Burt matrix, was analyzed (data not shown) (14). MCA was conducted, and the first two dimensions accounted for 64% (dimension 1 was 46% and dimension 2 was 18%) of Greenacre-adjusted inertia in the first two dimensions. The coordinates of the categorical variables in Figure 2 depicts the relationship between the values and categorical data, including the patient information. The position on the map of each categorical variable in Figure 2 shows the relationship between each variable, including the values and characteristics of the patients.

K-means cluster analysis was performed using object scores (data not shown) for dimensions 1 and 2, respectively, to which each category was assigned. The clustering results identified four clusters (CCC = −2.3). The 95% probability ellipses for each cluster were calculated from the object scores of the categorical variables included in each cluster and overlaid on the MCA map (Figure 2).

The cluster containing effectiveness_most was placed at the center of the map, and the three clusters safety_most, economical_most, and daily life_most and other_most were placed around it. The cluster safety_most included safety_multiple_yes, effectiveness_multiple_no, history of allergy or side effects caused by biologics or Janus kinase inhibitor_yes, history of allergy or side effects caused by drugs in general_yes, and inactive. The cluster economical_most included economic_multiple_yes, age_<65, drugs_<5, and biologics or Janus kinase inhibitor usage history_before_no. daily life_most and other_most were in the same cluster and included other_multiple_yes.

## Discussion

In this study, pharmacists and physicians collaborated to conduct SDM in patients with rheumatic disease, and we articulated the relationship of each value that patients considered important, as well as the relationship between the values and patient background. “Effectiveness” was the most important value, while “Safety,” “Economical,” “Daily life,” and “Other” were selected based on the background and experience of each patient. Therefore, drugs are not solely prescribed as per the evidence or the values of healthcare providers; however, patient values are important in the selection of therapeutic agents. The acceptance of SDM by the patients in our study was good, and patients were satisfied with the process. The continuity of treatment rate (Table 5) was monitored to determine whether SDM with pharmacist participation was clinically effective; although not directly comparable or statistically meaningful, it was better than that observed in a previous study (21).

Understanding and catering to patient preferences are associated with adherence and a good treatment response (22, 23). Pharmacists need to evaluate the patients’ drug therapies in clinical practice in terms of efficacy, safety, cost of health services (economy), and utilization of health services (necessity) (24, 25). In SDM in our usual clinical practice, necessity was further divided into two categories from the pharmacist’s perspective to obtain more specific views of the patient’s values: daily life and others. Thus, we classified the values related to patient decision-making into five categories. The five categories were effectiveness, safety, economics, daily life, and others. In this study, the relationship between these five values and the patient background was diagrammed in MCA and K-means cluster analysis to clarify their relationship. Effectiveness was at the center of patient values in rheumatic diseases, with other values as sub-values of effectiveness. In particular, safety was placed opposite efficacy. Effectiveness was considered a positive factor for patients; safety, economics, daily life, and others were considered negative factors; and positive and negative values appeared to conflict with each other. Depending on the effect of the limiting factor (such as these negative factors) on the patient’s values, the values that the patient considers important may shift from effectiveness to other values.

In terms of sex, women were on the border of the effectiveness and safety clusters on the map, and significantly more women chose safety in multiple-selected values. Thus, women were more likely to select safety. Women are more concerned about safety when switching from biologics to biosimilar drugs (26), which is also consistent with our results.

Patients who had experienced allergies or side effects in the past due to drugs for rheumatic diseases and/or other diseases tended to emphasize safety and showed deep concern about adverse effects after changing drugs. The results of MCA, in which the history of allergy or side effects caused by biologics or Janus kinase inhibitors (the main therapeutic agents for rheumatic diseases) was closer to the value of safety than to that of history caused by overall drugs the patient was taking, indicated that the priority of values shifts from effectiveness to safety after experiencing allergy or side effects caused by therapeutic agents for rheumatic diseases. However, the results may differ for other diseases. In rheumatic diseases, efficacy must be ensured first, followed by side effect management (27, 28). However, the value priorities of patients with malignant tumors and other diseases may differ from those of patients with autoimmune diseases due to different drug treatment intensities and frequency of allergy or side effects.

Age is one of the most important aspects of experience, and it is inferred that age influences patient values. In the results, the value with the highest ratio of patients <65 years in both multiple and single selections was economics, indicating a shift in the importance of the value from effectiveness to economics for younger patients. Younger patients generally chose intensive pharmacological treatment more frequently (29). Thus, we hypothesized that patients <65 years would select effectiveness; however, the result of the map indicated that the economic limitations of early age outweigh effectiveness. Younger patients have higher blood levels of tumor necrosis factor-α, while older patients have higher levels of interleukin-6, indicating a difference in signaling pathways in the pathogenesis of RA between juvenile RA and senile RA (30). In general, as age increases, physical deterioration due to aging and osteoarthrosis causes movement limitations, especially in RA, which are often accompanied by joint destruction and degeneration due to inflammation (31). Therefore, it was inferred that the values that patients consider important differ with age, and it was observed on the map that the values shifted with age from economics to the border between effectiveness and daily life.

It is important to discuss the economic issues of the patient regardless of the patient’s awareness and share the importance of these issues with the healthcare provider and patient for joint decision-making (32). Previous studies have indicated that biologics are more effective than conventional synthetic disease-modifying antirheumatic drugs such as methotrexate and salazosulfapyridine (27, 28). The usage history of biologics or Janus kinase inhibitors lies in the effectiveness cluster on the map, suggesting that patients with these histories experienced high treatment efficacy with these drugs and valued their effectiveness. However, these were not used in the economic cluster. These patients had no experience with treatment with highly effective but expensive drugs and were more concerned about economics than effectiveness, which suggested that their values shifted from effectiveness to economics. This may indicate that patients who should be treated with biologics or Janus kinase inhibitors do not have access to appropriate drugs due to a lack of experience with therapeutic efficacy.

The time spent on drug treatment and the actions related to the drugs themselves are included in daily life and others, which affect the physical and mental burden, leading to loss of patient productivity. For patients to continue drug treatment, this burden must be alleviated. Some patients prefer being managed by a healthcare provider and opt for intravenous infusion (33). Not preferring self-injection, less frequent administration, and preferring to be administered by a healthcare provider were factors in this outcome. However, in recent years, patient orientation has changed with the introduction of simple self-injection preparations, such as auto-injectors and oral small molecular targeted drugs (34). Recent studies have reported a preference for oral drugs over injections (33, 34), and a trend towards patient preference for oral drugs is becoming apparent. In our study, the route of administration chosen was different for each patient, but most patients took the drug orally, in addition to choosing routes such as intravenous infusion and subcutaneous injection (administered by themselves and nurses). In other words, this may mean that patients do not prioritize the route of administration in their effectiveness-centered values.

Thus, SDM has the potential to support the provision of tailor-made medical care that matches patient values. Biologics and Janus kinase inhibitors had similar efficacy on average and the same level of recommendation (27, 28). In the absence of clear drug superiority, there is room for interventions in decision-making based on patient values. In doing so, the healthcare provider needs to play a role in supporting decision-making in a non-paternalistic manner (35, 36).

There are some limitations to the study: this was a single-center study, the study population was small, and lifestyle-related diseases or other comorbidities and the type and route of administration of rheumatic disease medications were not included and considered as MCA items. Future studies with a larger sample size should examine the relationship between the values associated with each drug and the route of administration. The hospital where this study was conducted was a rural hospital with a high patient age range due to its regional characteristics, and the median age of the patients with rheumatic disease included in the study was as high as 66 [52–71] years. In addition, the duration of disease was 8 [3–16] years, and 43% of the patients had had the disease for more than 10 years. Patients with a wide range of ages and disease durations who needed SDM and participated in the SDM in the outpatient clinic were enrolled, and there was no selection bias. Since changes in life stage can affect the continuation of medications, the fact that a wide range of ages and durations of disease were adequately represented in the study is a strength of this study. Moreover, this was a pilot study involving pharmacists in the SDM of drug therapy for rheumatic diseases, which allowed us to accurately evaluate patients’ values and analyze their interrelationships at a single center.

Disease activity is a factor that influences treatment choice in the field of rheumatic diseases, as indicated in the treatment recommendations (27, 28). In the practice that we conduct according to treatment recommendations, we consider clinical remission to be the goal of medical care and tolerate low disease activity in some cases. Therefore, we classified patients into remission or low disease activity (Inactive) and moderate or high disease activity (Active) groups based on patient characteristics. Inactive patients used biologics or Janus kinase inhibitors before SDM more frequently than did Active patients, and their disease activity was controlled. They also rated therapeutic safety higher. Active patients were included in the same cluster as effectiveness; hence, we can say that disease activity is a factor that influences effectiveness and safety. However, there were no differences in other patient characteristics among disease activity, and values such as economical, daily life, and other seemed to be related to factors other than disease activity. Further research is needed to determine how disease activity influences other factors and values, as well as treatment choice.

## Conclusion

Even among patients with the same rheumatic disease, the subject experiences of patients with the treatment and/or the stage of life in which they were treated shaped the values they prioritized. Moreover, the relationships between each value affected the decision-making of patients regarding drug therapy. Patients make decisions based on multiple values rather than just one. There were values most influential to the patient that were important in decision-making; non-etheless, other associated values including patient background were also key factors. Since each patient has different values, the information that the pharmacist should provide as a healthcare provider may differ from the information wanted by the patient. To improve patient adherence and avoid the nocebo effect (37), it is important to positively link patient values and information about the treatment plan in SDM while establishing rapport (38) with the patient, rather than provide information based on the values of the healthcare provider.

## Data Availability

The original contributions presented in the study are included in the article/supplementary material, further inquiries can be directed to the corresponding author.
